# Human In Vivo Validation of Frequency‐Dependent QTI

**DOI:** 10.1002/mrm.70529

**Published:** 2026-08-13

**Authors:** Svenja Niesen, Marten Veldmann, Ali Ajouz, Tony Stöcker

**Affiliations:** ^1^ MR Physics, German Center for Neurodegenerative Diseases (DZNE) Bonn Germany; ^2^ Faculty of Mathematics and Natural Sciences University of Bonn Bonn Germany; ^3^ Department of Radiology and Neuroradiology UKSH, CAU Kiel Kiel Germany; ^4^ Section Biomedical Imaging, Department of Radiology and Neuroradiology University Hospital Schleswig‐Holstein, Christian‐Albrechts‐Universität zu Kiel Kiel Germany; ^5^ Siemens Healthineers AG Hamburg Germany; ^6^ Department of Physics & Astronomy University of Bonn Bonn Germany

**Keywords:** microstructure, QTI, spiral imaging, tensor‐valued encoding, time dependent diffusion

## Abstract

**Purpose:**

The aim of this work is to investigate if q‐space trajectory imaging (QTI) waveforms can be designed to probe QTI metrics at a single centroid frequency under realistic experimental conditions for in vivo human brain imaging.

**Methods:**

Realistic diffusion encoding waveforms based on double‐rotation gradient waveform and magic‐angle spinning of the q‐vector with varying encoding bandwidth, alignment of encoding spectra across encodings (tuning) and across axes (spectral isotropy) were designed to investigate the accuracy of the centroid frequency approximation. QTI metrics were computed for idealized as well as realistic oscillating and pulsed gradient waveforms using analytical diffusion spectra with 1D and 2D short‐range disorder along and perpendicular to axons. For in vivo validation, the waveforms were integrated into a multiband spiral spin‐echo sequence.

**Results:**

The simulations demonstrate frequency‐dependent QTI metrics. The combination of tuning and spectral isotropy led to metrics close to the simulated ground‐truth at the centroid frequency of the encoding spectrum. Realistic waveforms with broader bandwidth compared to idealized waveforms introduce little additional error in QTI metrics due to truncation of the cumulant expansion. In agreement with literature, omitting tuning and spectral isotropy reduces isotropic variance and increases microscopic fractional anisotropy when LTE contains lower frequencies, and vice versa for PTE. This observation is confirmed qualitatively by the in vivo measurements.

**Conclusion:**

The accuracy of the centroid frequency approximation depends on the combination of encoding spectra and investigated tissue. Using similar encoding spectra for tuning and spectral isotropy leads to metrics, characteristic to the centroid frequency.

## 1 Introduction

Diffusion tensor imaging [1, 2] enables investigation of the human brain structure beyond the resolution typically gained with MRI. The application of diffusion sensitizing gradients allows the definition of a voxel‐dependent diffusion tensor, that describes the averaged movement of molecules in that voxel and assumes this diffusion to be time‐independent (Gaussian diffusion). This allows the computation of macroscopic diffusion metrics such as the mean diffusivity (MD) and the fractional anisotropy (FA).

As Gaussian diffusion does not describe diffusion in complex tissue in the human brain sufficiently, other diffusion MRI (dMRI) methods emerged.

Oscillating gradients [3, 4, 5] were used to target diffusion times in the short‐time regime, yielding surface‐to‐volume ratios [6, 7], or the long‐time regime, in which time‐dependence results from the structural disordering of the tissue [8, 9, 10, 11, 12]. In particular, it was shown [8, 9, 10, 11, 12] that the one‐dimensional (1D) short‐range disorder along axons due to axon caliber variations as well as the two‐dimensional (2D) short‐range disorder of the extra‐axonal space perpendicular to the principal fiber orientation have a strong time‐influence on diffusion metrics.

Tensor valued encoding (TVE) [13] assumes a diffusion tensor distribution (DTD) composed of multiple Gaussian diffusion compartments within a voxel. Exploiting various b‐tensor shapes allows the computation of the DTD [14] and is achieved by shaping the diffusion gradients such that the desired q‐space trajectory is traversed.

However, due to the multiple unknowns arising from the components of the diffusion tensors in different compartments, many volumes with different diffusion encodings have to be acquired. Q‐space trajectory imaging (QTI) [15] offers a faster solution by the estimation of the covariance tensor of the DTD with a minimum of 28 diffusion volumes to compute microscopic metrics such as isotropic variance (Cmd) and microscopic fractional anisotropy (μFA). Each diffusion volume has to resemble a different b‐tensor with a specific b‐value and shape, which is defined by the b‐tensor's rank [15] (Rank 1: linear tensor encoding (LTE), Rank 2: planar tensor encoding (PTE), Rank 3: spherical tensor encoding (STE)).

Time‐dependent diffusion processes can be modeled with the Gaussian‐phase approximation [16], which leads to a frequency‐dependent DTD [17, 18, 19]. The complete quantification of the DTD requires different b‐tensor shapes designed to encode multiple frequencies. However, restricting the investigation to double‐rotation gradient waveforms at the centroid frequency [17, 18, 20]—the first moment of the encoding distribution—makes the approach applicable for in vivo measurements [21, 22].

Introducing time‐dependence in the TVE diffusional variance decomposition approach [23, 24, 25], it was demonstrated that matching the power spectra across different encodings (tuning) yields time‐dependent metrics that differ from those obtained without tuning. In that context, matching power spectra across axes within one encoding (spectral isotropy) [24] was introduced as well. Shifting the encoding spectra across frequencies results in time‐dependent MD estimates and altered μFA sensitivity, where the most affected frequencies depend on the compartment radius [25]. Monte‐Carlo simulations demonstrated that omitting tuning leads to overestimated μFA values using QTI, however to a less extent in white matter (WM) [26]. Additionally, time‐dependent μFA was shown with double (oscillating) diffusion encoding in both monkey [27] and mouse brains [28], including an error arising from overstepping the convergence radius [28]. Recently, matching the power spectra's trace across encodings was found to be essential to not over‐ or underestimate microscopic anisotropy due to different MD weighting across encodings in ex vivo rat heart [29]/brain [30] as well as in the prostate [31].

In this work, we follow up on the idea of Lasič et al. [30] by investigating if QTI waveforms for human brain MRI can be designed with encoding spectra narrowly distributed around the centroid frequency, such that the approximation of solely probing the centroid frequency can be used for deriving QTI metrics related to this frequency.

Different DTDs are simulated by using the analytical diffusion spectra [8, 9, 10, 11, 12] of the 1D and 2D short‐range disorder along and perpendicular to the axons (ω^0.5^ and ω^1^ power laws) as well as radial diffusion inside the axons (ω^2^ power law). Idealized oscillating and pulsed gradient echo waveforms (OGE and PGE) with varying centroid frequencies and spectral bandwidths are created to investigate under which conditions the ground‐truth QTI metrics can be measured at the centroid frequency. The bias due to this centroid frequency approximation is compared to the error arising from the cumulant expansion.

To investigate the practicality of the centroid frequency approximation, three QTI waveforms for in vivo human brain measurements are designed. Their encoding spectra differ, reflecting the use of tuning and spectral isotropy.

The three QTI waveforms are applied in vivo by incorporating them into a multiband spin‐echo sequence with a spiral readout. It is investigated if the simulation results correlate with the in vivo results.

## 2 Theory

The explanation of the centroid frequency approximation in QTI starts with a brief summary of the time‐independent QTI‐approach [15], which assumes a DTD that only includes Gaussian diffusion within each compartment. Here, the term “compartment” includes all particles whose diffusion can be described by the same diffusion tensor [14]. The time‐independent QTI‐approach [15] provides the signal attenuation for multiple Gaussian diffusion compartments:

(1) $$S\left(b\right)=S_{0}\cdot \left\langle \exp\left(-<b,d>\right)\right\rangle =S_{0}\cdot \sum_{i}w_{i}\cdot \exp\left(-<b,d_{i}>\right)$$

where **b** is the b‐tensor, **d**_i_ are the time‐independent diffusion tensors of the DTD, $S_{0}$ is the signal without diffusion weighting and $w_{i}$ is the weight of the i‐th compartment. Following the formalism of Westin et al. [15], <.,.> denotes the inner product between tensors and ⟨…⟩ the mean over compartments. The cumulant expansion of Equation (1) yields a signal attenuation depending on the averaged diffusion tensor ⟨d⟩ and a fourth order covariance tensor **C**, which leads to the time‐independent QTI‐equation [15]:

(2) $$S_{c}\left(b\right)=S_{0}\cdot \exp\left(-<b,\left\langle d\right\rangle >+\frac{1}{2}<B,C>\right).$$

Determination of **C** enables the computation of QTI metrics (MD, FA, Cmd, μFA) [15]. Measuring metrics with regards to time‐dependent diffusion within the compartments considers the Gaussian‐phase approximation for multiple compartments [16, 18]. Its Fourier domain representation is given by [18]:

(3) $$S\left(\omega \right)=S_{0}\cdot \sum_{i}w_{i}\cdot \exp\left(-\int_{-\infty }^{\infty }<b\left(\omega \right),d_{i}\left(\omega \right)>\text{d}\omega \right),$$

where **d**_i_(ω) is the tensor of the diffusion spectrum of the i‐th compartment and **b**(ω) is the encoding spectrum of the diffusion gradients $\vec{g}$ during the diffusion encoding time τ [17]:

(4) $$b\left(\omega \right)=\frac{1}{2\pi }\vec{q}\left(\omega \right)⊗\vec{q}\left(\omega \right)$$

with the outer product ⊗ and the dephasing spectrum $\vec{q}\left(\omega \right)$ defined as:

(5) $$\vec{q}\left(\omega \right)=\int_{0}^{\tau }\left(\gamma \int_{0}^{t}\vec{g}\left(t^{\prime }\right){\mathrm{dt}}^{\prime }\right)e^{\mathrm{i\omega t}}\mathrm{dt}.$$

Equation (3) can be simplified to Equation (1) if **d**(ω) is constant across ω for nonzero **b**(ω) [18]. In this work, we investigate whether the simplification to Equation (1) remains valid when **b**(ω) has nonzero values narrowly distributed around the centroid frequency ω_c_, such that **d**(ω) = **d**(ω_c_). The centroid frequency ω_c_ is defined as [17, 20]:

(6) $$\omega_{c}=\frac{1}{b}\int_{-\infty }^{\infty }\left|\omega \right|\;b\left(\omega \right)\;d\omega .$$

Here, b(ω) is the frequency‐dependent trace of **b**(ω) and b is the integral of b(ω) across frequencies. Thus, Equation (3) can be simplified to:

(7) $$S\left(\omega_{c}\right)=S_{0}\cdot \sum_{i}w_{i}\cdot \exp\left(-<b,d_{i}\left(\omega_{c}\right)>\right).$$

The cumulant expansion of S(ω_c_) is denoted as S_c_(ω_c_) as in Equation (2), but the averaged diffusion tensor ⟨d⟩ and the covariance tensor **C** now depend on ω_c_:

(8) $$S_{c}\left(\omega_{c}\right)=S_{0}\cdot \exp\left(-<b,\left\langle d\left(\omega_{c}\right)\right\rangle >+\frac{1}{2}<B,C\left(\omega_{c}\right)>\right).$$

Estimation of **C**(ω_c_) leads to QTI metrics specific for the centroid frequency.

## 3 Methods

### 3.1 Frequency Modulated QTI Waveforms

To investigate the validity of the centroid frequency approximation, idealized oscillating and pulsed gradient waveforms (OGE and PGE) with varying centroid frequency and spectral bandwidth were designed. The OGE waveforms (Figure 1A) were created with trapezoidal gradients of alternating polarity. The first and last trapezoidal gradients had duration τ, while gradients in the center of the train had duration 2τ. The duration τ was varied to create waveforms with frequencies ranging from 1 to 21 Hz. Increasing the number of gradients in the middle of the train decreases the spectral bandwidth, defined here as the frequency interval containing 90% of the total area under the encoding spectrum. The PGE waveforms (Figure 1B) were created with the gradient duration δ fixed at 12.2 ms and varying the gradient spacing Δ. Increasing the spacing increases both ω_c_ and spectral bandwidth.

**FIGURE 1:**
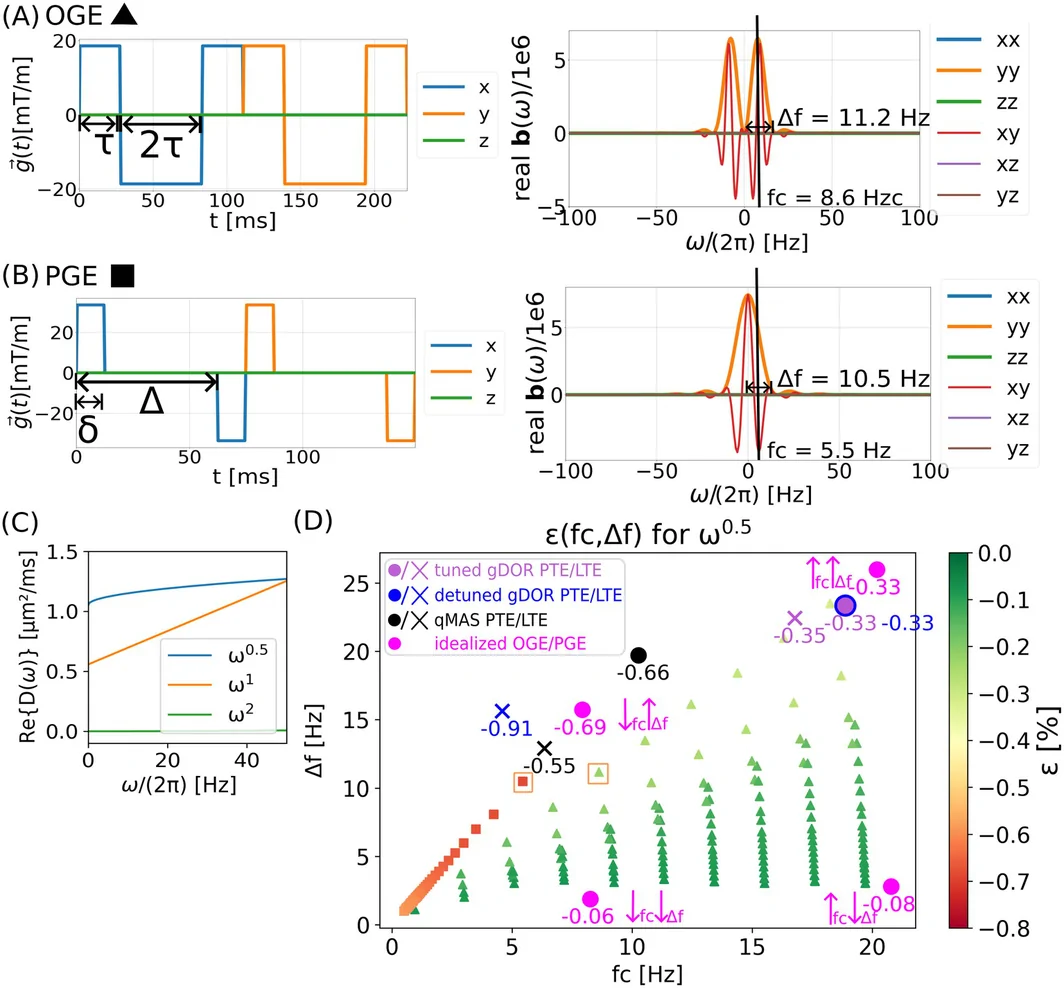
(A) Idealized oscillating gradient echo (OGE) waveform with τ as the duration of the first trapezoidal gradient, defining the centroid frequency f_c_ in b(ω) and the number of gradient oscillations defining the spectral bandwidth Δf in b(ω). (B) Idealized pulsed gradient echo (PGE) waveform with δ as the duration of the trapezoidal gradient and Δ as the spacing between the two trapezoidal gradients. (C) The real part of the diffusion spectrum behaves as ω^0.5^ for the 1D short‐range disorder along axons [8, 9, 10], as ω^1^ for the 2D short‐range disorder perpendicular to the axons in the extra‐axonal space [8, 9, 11, 12], and as ω^2^ for the radial diffusivity inside the axons [11, 16]. (D) Relative error ε (Equation 14): The effective diffusivity differs more from the diffusivity at the centroid frequency f_c_ with decreased centroid frequency f_c_ and increased spectral bandwidth Δf. The waveforms of (A) and (B) are circled by a rectangular shape with squares demonstrating PGE and triangles demonstrating OGE waveforms. The errors of the in vivo waveforms match the errors obtained from the idealized OGE and PGE waveforms at the respective centroid frequency and bandwidth. The four combinations of the idealized sequences, shown in Table 1, are presented as pink dots.

To compute QTI metrics, four waveforms were selected with low/high ω_c_ and low/high spectral bandwidth as indicated by the arrows in Table 1A. PTE waveforms (arrows with solid line) were designed by applying the OGE or PGE waveform on the x‐axis and y‐axis, while application solely on the z‐axis created the LTE (arrows with dashed line). These four waveforms were combined to 16 permutations (4 PTE · 4 LTE).

**TABLE 1:** (A) The four idealized OGE and PGE waveforms that are combined for QTI sequence investigation. A high or low centroid frequency f_c_ is indicated by the first arrow pointing up or down and the same holds for the second arrow for the spectral bandwidth Δf. PTE waveforms are marked with solid lines and LTE waveforms with dashed lines. Here, only 4 out of the 16 combinations with varying PTE and LTE frequencies are shown. The four combinations are illustrated as pink dots in Figure 1D. (B) The centroid frequencies and bandwidths for the three in vivo sequences are shown, for both PTE and LTE waveforms and their combination (mean of PTE and LTE volumes).

	f_c_ [Hz]	Δf [Hz]
(A) Idealized sequences		
PTE: ↓↓; LTE: ⇣⇣	8.2	1.9
PTE: ↓↑; LTE: ⇣⇡	7.9	15.7
PTE: ↑↓; LTE: ⇡⇣	20.8	2.8
PTE: ↑↑; LTE: ⇡⇡	20.2	26.0
(B) In vivo sequences		
Tuned gDOR (PTE/LTE/combined)	18.9/16.8/17.4	23.4/22.4/22.7
Detuned gDOR (PTE/LTE/combined)	18.9/4.6/8.9	23.4/15.6/17.9
qMAS (PTE/LTE/combined)	10.3/6.4/7.6	19.7/12.9/14.9

Additionally, three QTI waveforms suitable for human brain measurements within scanner hardware limits were designed. To better distinguish them from the idealized OGE and PGE waveforms, these are hereinafter referred to as oscillating and pulsed gradient spin‐echo waveforms (OGSE and PGSE). As STE waveform design could not ensure spectral isotropy, PTE and LTE were used.

Figure 2A shows the “tuned gDOR” [17, 18] waveforms with spectral isotropy on PTE and tuned LTE. MATLAB's genetic algorithm [34, 35] was used to find the one‐dimensional g1d(t) waveform from double‐rotation gradient waveform (gDOR) [17, 18], which was designed here with a positive and negative trapezoidal gradient before the refocusing pulse (Table S1). The amplitudes, ramps and flat‐top times of the trapezoids and the delay between the trapezoids were optimized as follows: For the PTE waveform, the objective contained matching spectra for the x‐ and y‐axis with **b**(ω) being centered closely around ω_c_/(2π) = 18 Hz (Table 1B). The optimized PTE waveform shows strong similarity to the previously published circular polarized OGSE sequence [36] (Figure S1A,B). Ideally, a single peak would appear at ω_c_. However, a second peak is observed in the encoding spectra due to a finite duration of the refocusing pulse and crusher gradients [28]. The magnitude of this secondary peak is proportional to the ratio of refocusing duration and TE. As the refocusing duration was limited by SAR and peripheral nerve stimulation due to the crushers and TE was limited by T2 decay, the second peak could not be fully eliminated. It is tested, if the splitting into two peaks leads to the same errors in the centroid frequency approximation as for an OGSE sequence with same waveform duration, ω_c_ and bandwidth, but establishing from one peak (Figure S1A,C). The LTE waveform was optimized to match the encoding spectrum of the PTE considering the trace of the PTE waveform and its centroid frequency (Table 1B). Nonlinear constraints were imposed to prevent exceeding the scanner's maximum gradient amplitude and slew rate per axis, while ensuring the desired b‐tensor shape [14], the maximum b‐value, the spin‐echo condition and no diffusion gradients during the refocusing duration. A linear constraint guaranteed the target waveform duration.

**FIGURE 2:**
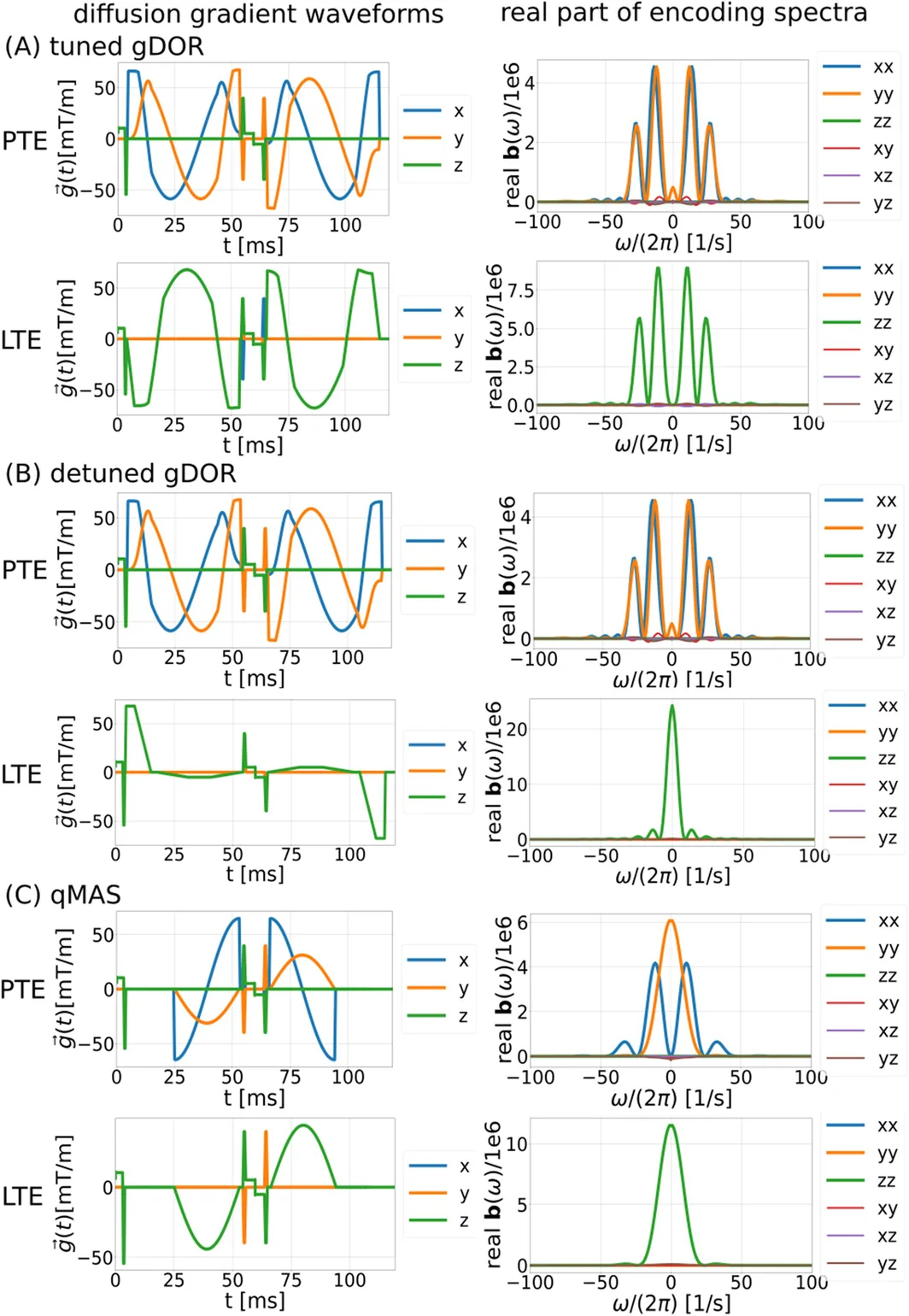
(A–C) First column: Optimized diffusion gradients for the double‐rotation gradient waveform (gDOR) [17, 18] and the corrected magic‐angle spinning of the q‐vector (qMAS) [32, 33] sequence. Slice‐selection and crusher gradients are illustrated as they were incorporated into the b‐tensor calculation. Second column: Real part of the encoding spectrum b(ω).

The same g1d(t) waveform parameters were used for the LTE waveform of “detuned gDOR”, but the objective was to center **b**(ω) closely around 0 Hz (Figure 2B and Table 1B) to differ from PTE's encoding spectrum (detuning). All other constraints matched those of “tuned gDOR”. The optimized parameters for the gDOR sequences are listed in Table S1.

Figure 2C shows magic‐angle spinning of the q‐vector (qMAS) waveforms [32, 33]. As done for finite refocusing duration in Ref. [33], correction factors were computed to consider a finite slew rate and applied to the gradient amplitudes. Using MATLAB's “fmincon” algorithm [34, 37], STE waveform parameters were optimized (Table S2) to minimize TE under the given scanner limits, and two (one) STE gradient axes were used for PTE (LTE). Thus, the PTE y‐axis spectrum fits the LTE z‐axis spectrum (tuning) with the PTE spectrum containing spectral anisotropy (Table 1B).

For each sequence, a crusher moment of 4π along each axis was ensured for each diffusion volume to suppress unwanted signal originating from the refocusing pulse (Figures S2–S4).

### 3.2 Centroid Frequency Approximation on 1D Diffusion Spectra

To investigate the error of the centroid frequency approximation, dependent on ω_c_, spectral bandwidth and tissue, Equation (3) is reduced to a 1D case:

(9) $$S_{1D}\left(\omega \right)=S_{0}\cdot \exp\left(-\int_{-\infty }^{\infty }b\left(\omega \right)\cdot d\left(\omega \right)\;\text{d}\omega \right).$$

The encoding spectra's trace b(ω) was computed with Equations (4) and (5) for the idealized PTE waveforms (OGE and PGE) and the waveforms for in vivo measurement.

The tissue‐influence on the approximation was explored by setting the real part of the diffusion spectrum d(ω) to (Figure 1C):

1. Diffusion along the axons as 1D short‐range disorder [8, 9, 10]:
(10) $$\text{Real}\left\{d\left(\omega \right)\right\}=D_{\infty }+\frac{c2}{2}\cdot \frac{\sqrt{\pi }}{2}{\left|\omega \right|}^{0.5},$$
  with $D_{\infty }$= 1.054 μm^2^/ms and c2 = 0.616 (μm^2^/ms)^0.5^ for the anterior corona radiata (ACR) region [38].
2. Diffusion perpendicular to the axons in the extra‐axonal space as 2D short‐range disorder [8, 9, 11, 12]:
(11) $$\text{Real}\left\{d\left(\omega \right)\right\}=D_{\infty }+A\cdot \frac{\pi }{2}{\left|\omega \right|}^{1},$$
  with $D_{\infty }$= 0.558 μm^2^/ms and A = 1.410 μm^2^ for the ACR region [38].
3. Diffusion perpendicular to the axons in the intra‐axonal space as the long‐time regime inside a perfectly reflecting cylinder [11, 16]:
(12) $$\text{Real}\left\{d\left(\omega \right)\right\}=\frac{7}{96}+\frac{a^{4}}{D_{0}}\cdot {\left|\omega \right|}^{2},$$
  with $D_{0}$ = 2 μm^2^/ms and the radius a = 1.20 μm.

For each diffusion spectrum, S_1D_(ω) was computed with insertion of the encoding spectra into Equation (9) and S_0_ being set to one. The effective diffusivities d_eff_ were determined by:

(13) $$d_{\mathrm{eff}}=\frac{\ln\left(S_{1D}\left(\omega \right)\right)}{-b}$$

with the b‐value b. The relative error:

(14) $$\varepsilon =\frac{\left(d_{\mathrm{eff}}-d\left(\omega_{c}\right)\right)}{d\left(\omega_{c}\right)}\cdot 100,$$

was reported for each encoding spectrum.

### 3.3 Centroid Frequency Approximation on 3D Diffusion Spectra for QTI

The introduced 1D diffusion spectra were combined yielding four different DTDs (Figure 3A):

1. One isotropic diffusion tensor with diffusivity following the power law ω^1^ (Equation 11).
2. One anisotropic diffusion tensor with radial diffusivity following the power law ω^1^ (Equation 11) and the axial diffusivity following the power law ω^0.5^ (Equation 10).
3. The anisotropic tensor from case 2 and diffusion tensors with radial diffusivity following the power law ω^2^ (Equation 12) and axial diffusivity ω^0.5^ (Equation 10), resembling parallel fibers with an axonal volume fraction of 0.5. The radius distribution was taken from the histogram of radii in Ref. [11] (fig. 11 in Ref. [11], Sector 6 of rhesus monkey corpus callosum).
4. The DTD of case 3, but the diffusion tensor, representing the parallel fibers, was rotated by 45° around the y‐axis resembling a first crossing fiber and an additional rotation by 180° around the z‐axis resembled the second crossing fiber. The DTD was sought to simulate the ACR.

**FIGURE 3:**
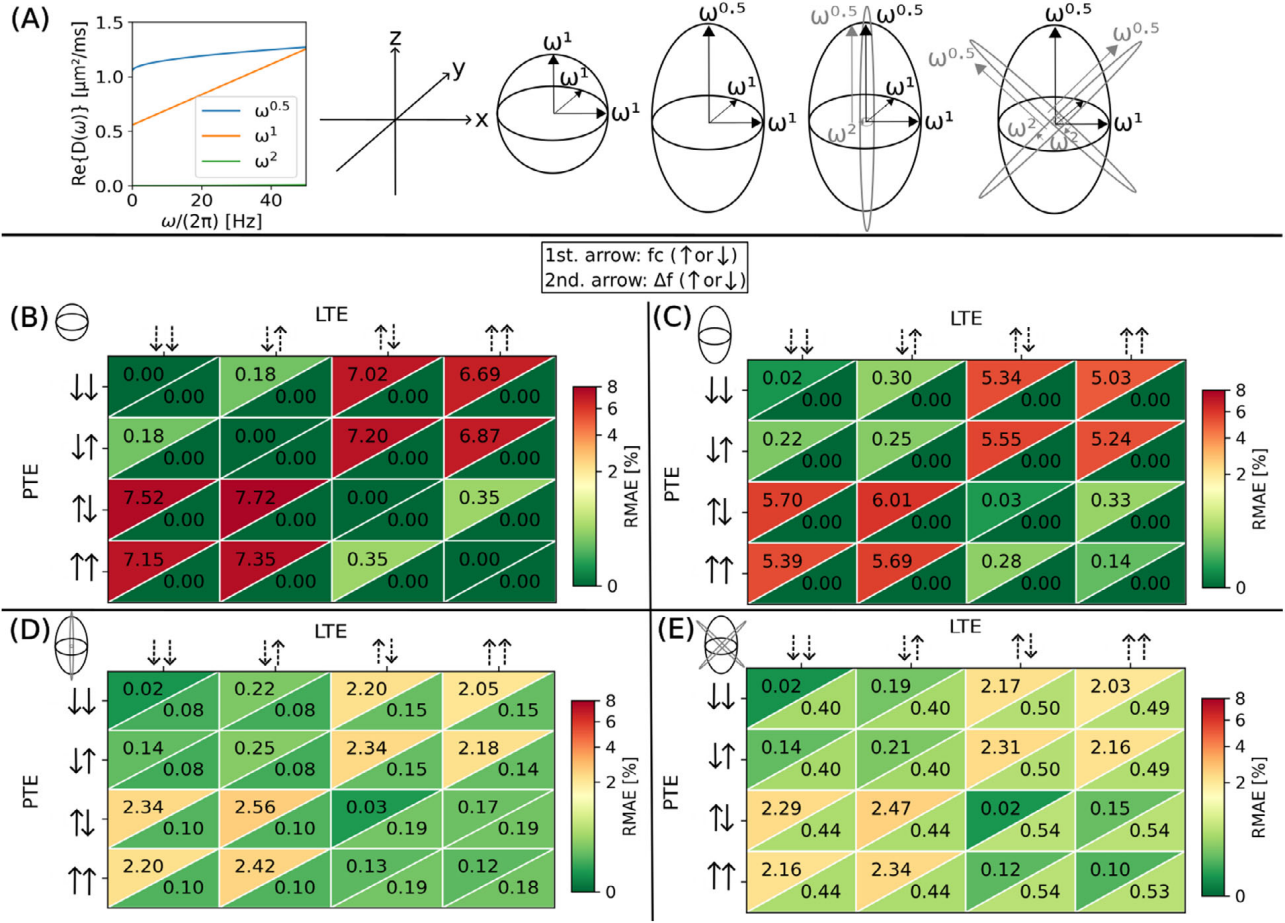
(A) Four different frequency‐dependent diffusion tensor distributions were created from the one‐dimensional diffusion spectra shown in Figure 1C: An isotropic tensor, an anisotropic tensor, an anisotropic tensor for the extra‐axonal space and parallel fibers and crossing fibers, with an axonal volume fraction of 0.5 for the last two distributions. (B–E) Relative mean absolute errors (RMAE) due to the centroid frequency approximation RMAE(S(ω), S(ω_c_)) (upper triangle) and truncation error in the cumulant expansion RMAE(S(ω_c_), S_c_(ω_c_)) (lower triangle) are shown in a heat map for the 16 combinations of the idealized waveforms from Table 1 (pink markings in Figure 1D). The first arrow defines f_c_, the second Δf, the solid lines mark PTE and the dashed lines LTE waveforms. If PTE and LTE waveforms sample the same frequencies (main diagonal), the error of approximation increases with larger bandwidth if ω^0.5^ diffusion behavior is included in the tensor distribution. If PTE and LTE waveforms instead sample different frequencies, the error of approximation is strongly increased. The error from truncation of the cumulant expansion increases with increasing diffusivity differences in the tensor distribution.

Each of the derived DTDs was inserted into Equations (3), (7), and (8) with the non‐diffusion weighted signal S_0_ set to one. The gradients of each QTI sequence were inserted into Equations ((4), (5), (6)) to compute the encoding spectra and ω_c_. The resulting signals were computed with Equations (3), (7) and (8). For sequences with different ω_c_ for PTE and LTE, the mean centroid frequency was used as ω_c_ in Equations (7) and (8), hereafter referred to as combined frequency.

Signals (S_1_ and S_2_) were compared with the relative mean absolute error (RMAE) [39]:

(15) $$\text{RMAE}\left(S_{1},S_{2}\right)=\frac{100}{\mathrm{NA}}\sum_{n}^{\mathrm{NA}}\frac{\left|S_{2}(n)-S_{1}(n)\right|}{\left|S_{1}(n)\right|}$$

where NA is the number of diffusion volumes. To investigate if **b**(ω) was narrowly distributed around ω_c_ to measure **d**(ω_c_), S(ω) (Equation 3) was compared with S(ω_c_) (Equation 7). Comparing S(ω_c_) (Equation 7) with S_c_(ω_c_) (Equation 8) investigated the bias arising from the truncation of the cumulant expansion. For parallel fibers, this was also done using only LTE volumes with gradient directions having an inner product below 0.3 with the main fiber orientation.

For the crossing fiber DTD, $\left\langle d\left(\omega_{c}\right)\right\rangle$ was computed as the mean over the tensors at ω_c_ and $C\left(\omega_{c}\right)$ was computed by inserting the DTD at ω_c_ into equation 6 in Ref. [15], leading to ground‐truth QTI metrics specific for ω_c_.

Taken the signals S(ω) and the b‐tensors as input, QTI metrics were calculated with DIPY [40] using the OLS solver and the SDPdc solver. Last imposes positivity on **d** and **C** [41].

To investigate the approximation error, the relative error (RE) between the ground‐truth QTI metrics, specific for ω_c_, and the calculated metrics for each sequence was computed.

Last step was repeated for the DTD with one anisotropic tensor.

### 3.4 In Vivo Experiments

#### 3.4.1 Sequence Parameters

Four healthy subjects (gender: four males, one female; age: 24–33) were scanned with given consent. Subject one was measured twice with a break of 8 days in between.

Measurements were conducted on a MAGNETOM 7T Plus scanner (Siemens Healthineers AG, Forchheim, Germany) with a 32/8 channel receive/transmit coil (Nova Medical), a maximum gradient strength of 70 mT/m and a maximum slew rate of 200 T/m/s.

The three QTI waveforms were inserted into a single‐shot spiral spin‐echo sequence [42, 43]. Sequence design was done with Pulseq [44] and the sequence parameters were: matrix size = 140 × 140, slices = 70, resolution = 1.5 mm isotropic, volume TR = 5.46 s, TE = 119.2 ms, total acquisition time = 15 min, in‐plane acceleration = 3, multiband‐factor = 2, acquired in transversal orientation. The Q_2_ scheme [45] was used as the diffusion protocol with a total amount of 151 diffusion volumes: encodings = [PTE, LTE], b‐values = [[0, 1400], [0, 100, 800, 1400]] s/mm^2^, directions per b‐values = [[9, 36], [22, 6, 30, 48]]. The excitation pulse duration was set to 6 ms and the refocusing pulse duration to 8 ms, leading to a total refocusing duration of 11.5 ms due to crushers and a 1 ms pause to decrease peripheral nerve stimulation. Fat unfocusing was achieved by adjusting the excitation and refocusing pulse bandwidths [46]. Additionally, a fat saturation pulse before the excitation pulse with a flip angle of 70° was applied.

A T1‐weighted (T1w) image was acquired with an MPRAGE sequence (FOV = 256 × 256 × 179.2 mm^3^; isotropic resolution = 0.8 mm, total acquisition time = 4 min) to enable a region of interest analysis in structural space.

#### 3.4.2 Reconstruction & Preprocessing Pipeline

The reconstruction workflow [42, 43] used a higher order encoding model [47], which included a B0 map, coil sensitivity maps (both derived from a GRE‐prescan), dynamic field monitoring [48] data of up to 3rd spatial order and compressed sensing regularization [49, 50].

Preprocessing included denoising [51], Gibbs ringing correction [52], motion correction with FSL “eddy” [53, 54, 55], gradient unwarping [56, 57] and a correction for thermal drift [58, 59, 60]. The thermal drift correction [60] was modified to apply region‐wise signal drift correction [61] with 10 k‐means clustered regions from a mean non‐diffusion weighted image. QTI metric computation was done with the QTI ± algorithm [41, 62, 63] with an upper diffusion limit of 3.1μm^2^/ms, used for the algorithm [41, 62, 63]. The b‐tensor calculation included imaging gradients and considered the systems response by applying the gradient transfer function (GTF) [64, 65] to the gradient waveforms. We assessed potential vibration‐induced bias in diffusion metrics by analyzing LTE signals at the maximum b‐value across b‐vector components for each QTI sequence as done in literature [66].

Registration [67, 68, 69] from diffusion space to structural space was done with the mean of the non‐diffusion weighted images and afterwards applied onto the QTI metric maps. The FSL MNI152‐template [67] was registered to structural space and region of interest analyses were performed for the splenium and the anterior corona radiata, by applying the registration from MNI to structural space on the labeled regions from FSL atlases [70, 71]. Boxplots containing the metric values within the two regions were computed for each subject and sequence. A linear mixed‐effects model with random intercepts [72] for subjects was fitted to assess the effect of the different sequences on the measured QTI metrics. Absolute and relative differences between the sequences were estimated from the fitted model and reported together with their corresponding 95% confidence intervals.

## 4 Results

### 4.1 Centroid Frequency Approximation on 1D Diffusion Spectra

The relative error ε (Equation 14) vanishes for 2D short‐range disorder (power law ω^1^) for each encoding spectra, whereas diffusion itself vanishes for movement perpendicular to and inside an axon (power law ω^2^) (Figure 1C).

1D short‐range disorder (power law ω^0.5^) demonstrates lower d_eff_ than d(ω_c_) with increasing bandwidth and decreasing ω_c_ of the idealized PGE/OGE waveforms (Figure 1D). The PGE spectrum with the largest bandwidth has the largest error (ε ≈ −0.8%). The error behavior of the idealized waveforms also holds for the in vivo waveforms (Figure 1D). The in vivo PGSE waveform error falls within the PGE range (largest error for LTE of qMAS and detuned gDOR), while the OGSE waveform error falls within the OGE range (smallest error for PTE of tuned and detuned gDOR). The PTE of qMAS, combining PGSE and OGSE, lies between both ranges and shows the second‐largest error.

### 4.2 Centroid Frequency Approximation on 3D Diffusion Spectra for QTI

The signal comparison between S(ω) and S(ω_c_) shows accurate centroid frequency approximation for an isotropic tensor, if PTE and LTE contain the same ω_c_ regardless of the bandwidth (Figure 3B). In contrast, the anisotropic tensor or multiple tensors show increasing RMAE with larger spectral bandwidth but the same ω_c_ (Figure 3C–E).

Higher RMAE is observed if PTE and LTE differ in ω_c_ (Figure 3B–E), especially for the isotropic tensor with diffusivity following the power law ω^1^. If diffusion waveforms contain larger frequencies than the combined frequency of all volumes, the signal level is smaller than the signal at the combined frequency (LTE/PTE volumes in the orange/purple marked cases in Figure 4A) and vice versa (PTE/LTE volumes in the orange/purple marked case in Figure 4A).

**FIGURE 4:**
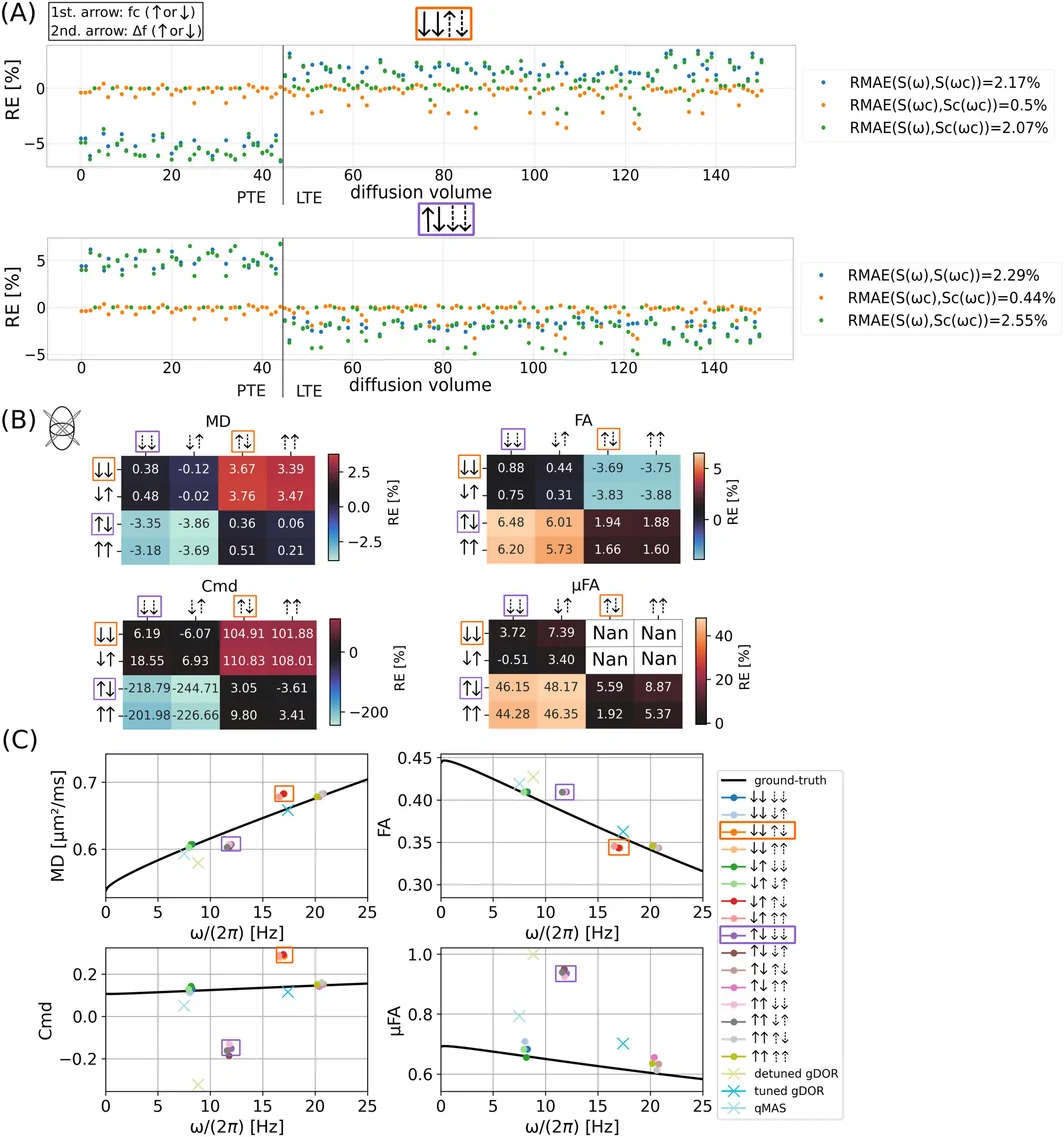
(A) For the crossing fiber diffusion tensor distribution (Figure 3A), the relative error (RE) with regards to the signals S(ω), S(ω_c_) and S_c_(ω_c_) is plotted over the 151 diffusion volumes for two PTE/LTE combinations. The relative mean absolute error (RMAE) is shown in the legend on the right side for each signal comparison. In the orange example shown, PTE/LTE waveforms have smaller/larger frequencies than the combined frequency of PTE and LTE waveforms. Therefore, S(ω_c_) is smaller than S(ω) for the PTE volumes as the MD is larger at the combined frequency, and the other way around for the LTE volumes. The purple example illustrates the reversed case with PTE/LTE waveforms having larger/smaller frequencies than the combined frequency of PTE and LTE waveforms. (B) The relative error (RE) for each metric, computed from S(ω), and the ground‐truth metric at the combined frequency is displayed as a heat map, using the arrows for f_c_ and Δf to differentiate between the 16 combinations of the idealized sequences. (C) The ground‐truth metrics are displayed as a black solid line and the metrics of the QTI sequences as dots for the 16 combinations of the idealized sequences and as crosses for the in vivo sequences. The mean of the PTE and LTE signal levels determines the over‐ or underestimation of the MD and the difference of the signal levels leads to overestimated Cmd and underestimated (undefined) μFA in the orange marked combination and to underestimated Cmd and overestimated μFA in the purple marked combination. To demonstrate directly the impact of the signal level on the QTI computation, Cmd and μFA step outside their boundaries ([0, 1]) as no constraints were imposed to the ordinary least squares solver in DIPY [40].

The truncation of the cumulant expansion causes no additional error for a single tensor (Figure 3B,C), but multiple tensors in the DTD (Figure 3D,E) show an increased truncation error at higher frequencies. The truncation error also increases when only LTE volumes with gradient directions perpendicular to parallel fibers are compared (Figure S5).

Between the in vivo sequences tuned gDOR has the lowest error due to the centroid frequency approximation, followed by qMAS and detuned gDOR (Table 2). For the truncation error, this order is reversed.

**TABLE 2:** (A) Left: For each QTI waveform, used for in vivo measurement, the relative mean absolute errors (RMAE) between the Gaussian‐phase approximated signal S(ω) and the signal defined by centroid frequency metrics S(ω_c_) as well as the RMAEs between S(ω_c_) and the cumulant signal S_c_(ω_c_) is presented. Right: The absolute values of the QTI metrics (MD: mean diffusivity, FA: fractional anisotropy, Cmd: isotropic variance, μFA: microscopic fractional anisotropy) and the relative error (RE) with regards to the ground‐truth metrics at the combined frequency of the corresponding waveform. Tuned gDOR shows lowest RMAE in the signal comparisons and lowest RE in the QTI metrics comparison, followed by qMAS and lastly detuned gDOR. (B) Left: Mean and standard deviation (SD) across subjects for each QTI waveform and metric. Right: Displayed are the absolute and relative difference as well as the confidence interval for each of the three waveform comparisons with the linear mixed‐effects model. Mean and standard deviation demonstrate the highest MD for tuned gDOR, followed by detuned gDOR and qMAS as well as small relative difference regarding FA and large relative differences with thin confidence interval between the gDOR sequences for Cmd and μFA.

(A) Simulated ACR	RMAE (S(ω), S(ωc)) [%]	RMAE (S(ωc), Sc(ωc)) [%]	MD [μm^2^/ms]/RE [%]	FA/RE [%]	Cmd/RE [%]	μFA/RE [%]
tuned gDOR	0.39	0.5	0.66/−0.34	0.36/2.27	0.12/−18.0	0.70/13.99
detuned gDOR	2.91	0.4	0.63/4.01	0.40/0.04	0.01/−94.0	0.91/38.21
qMAS	0.81	0.47	0.60/−0.79	0.42/1.3	0.06/−52.0	0.79/19.12

	Mean ± SD			Absolute difference		
				Relative difference [%]		
				95% confidence interval (relative difference [%])		
(B) ACR in vivo	Tuned gDOR	Detuned gDOR	qMAS	qMAS > detuned gDOR	Tuned gDOR > detuned gDOR	qMAS > tuned gDOR
MD	0.93 ± 0.03 [μm^2^/ms]	0.86 ± 0.04 [μm^2^/ms]	0.79 ± 0.03 [μm^2^/ms]	−0.06 [μm^2^/ms] −7.40 [−7.55, −7.26]	0.07 [μm^2^/ms] 8.33 [8.18, 8.47]	−0.14 [μm^2^/ms] −16.99 [−17.30, −16.68]
FA	0.41 ± 0.03	0.42 ± 0.02	0.42 ± 0.02	0.007 1.63 [1.40, 1.86]	−0.002 −0.46 [−0.69, −0.23]	0.009 2.06 [1.61, 2.51]
Cmd	0.18 ± 0.03	0.10 ± 0.03	0.15 ± 0.02	0.04 40.71 [39.97, 41.45]	0.07 70.70 [69.96, 71.43]	−0.03 −21.31 [−22.36, −20.26]
μFA	0.73 ± 0.03	0.88 ± 0.03	0.83 ± 0.01	−0.04 −5.00 [−5.10, −4.91]	−0.15 −17.18 [−17.28, −17.09]	0.11 12.82 [12.63, 13.02]

The ground‐truth QTI metrics of the simulated ACR (Figures 4C and 5B) show an increase in MD and Cmd and a decrease in FA and μFA with increasing frequency. The FA values are one‐half of the μFA values.

**FIGURE 5:**
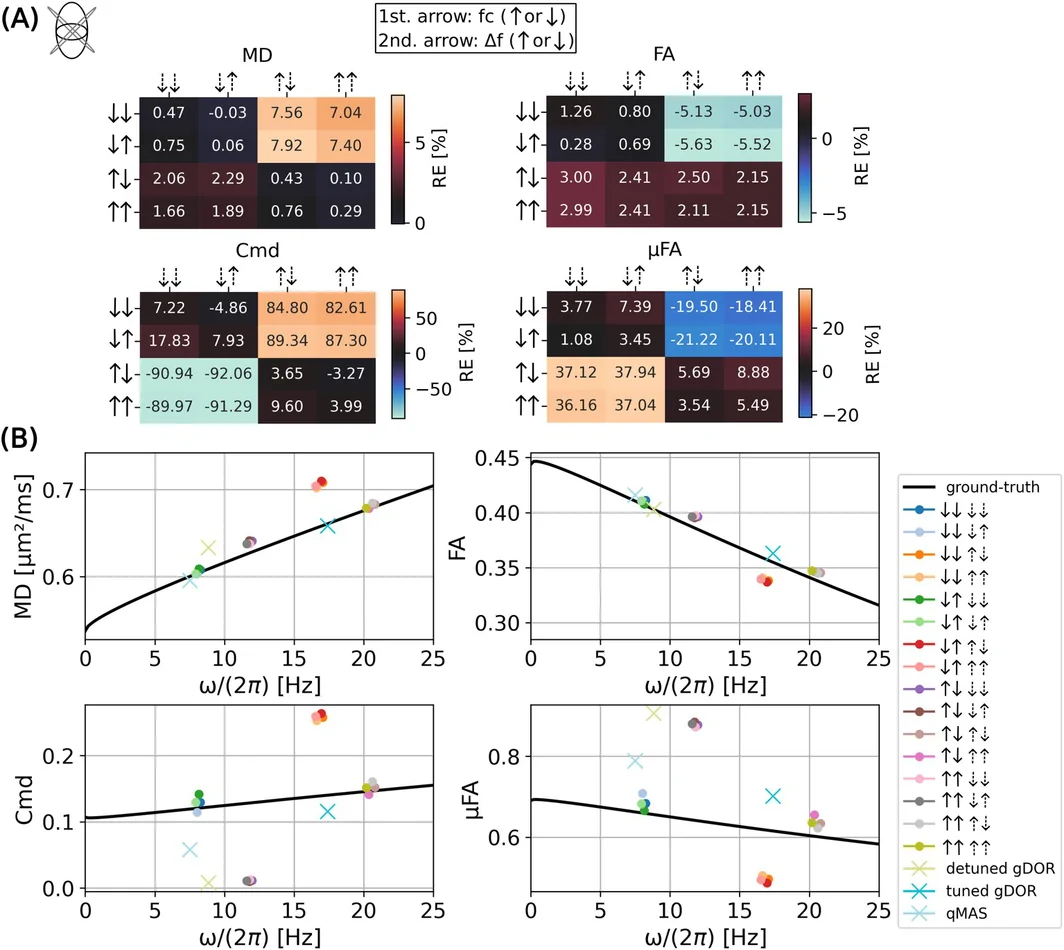
(A, B) Analogously to Figure 4B,C, but imposing positivity constraints on **d** and **C** in the QTI solver in DIPY [40]. As a result, negative values of Cmd without constraints (Figure 4C) are set close to zero and μFA values are no longer undefined. The bias between the metrics of the in vivo sequences and the ground‐truth metrics is the smallest for tuned gDOR, followed by qMAS and lastly by detuned gDOR. Last leads to strongly underestimated Cmd and overestimated μFA as the purple marked combination (Figure 4C).

For all QTI sequences (Figures 4B,C and 5), lower RE is found for the macroscopic metrics (MD and FA) than for the microscopic metrics (μFA and Cmd). Sequences with similar ω_c_ for PTE and LTE stay below a RE of 8% for all metrics of the simulated ACR. In contrast to a single anisotropic tensor in the DTD (Figure S6), the simulated ACR shows no increased RE with increased spectral bandwidth (Figures 4B,C and 5). Low RE is visible for tuned gDOR, but slightly higher than for sequences with similar ω_c_ for PTE and LTE (Table 2A).

Sequences with higher frequencies in PTE than in LTE spectra (purple markings in Figure 4) underestimate MD, overestimate FA, strongly underestimate Cmd up to negative values, and strongly overestimate μFA. Positivity constraints on **d** and **C** lead to Cmd values close to zero and μFA values close to one (Figures 4B,C and 5). Sequences with higher frequencies in LTE than in PTE spectra (orange markings in Figure 4) show the exact opposite behavior leading to an undefined μFA in the unconstrained case. The strong underestimation of Cmd and overestimation of μFA is apparent for detuned gDOR and qMAS (to a smaller extent) in contrast to the FA (Table 2A). Tuned gDOR shows the highest MD, followed by detuned gDOR and qMAS with low RE for all waveforms (Table 2A).

### 4.3 In Vivo Experiments

Figures 6 and 7 show MD, FA, Cmd and μFA for all QTI sequences. The MD is slightly larger for tuned gDOR than for detuned gDOR and qMAS across the WM, whereas for the FA there is no strong difference observed between the sequences. The Cmd/μFA decreases/increases from tuned gDOR over qMAS to detuned gDOR across the whole brain, as predicted by the simulation results (Table 2A). The region of the pallidum and caudate (red arrows) shows low MD, high Cmd and high (μ)FA for all sequences.

**FIGURE 6:**
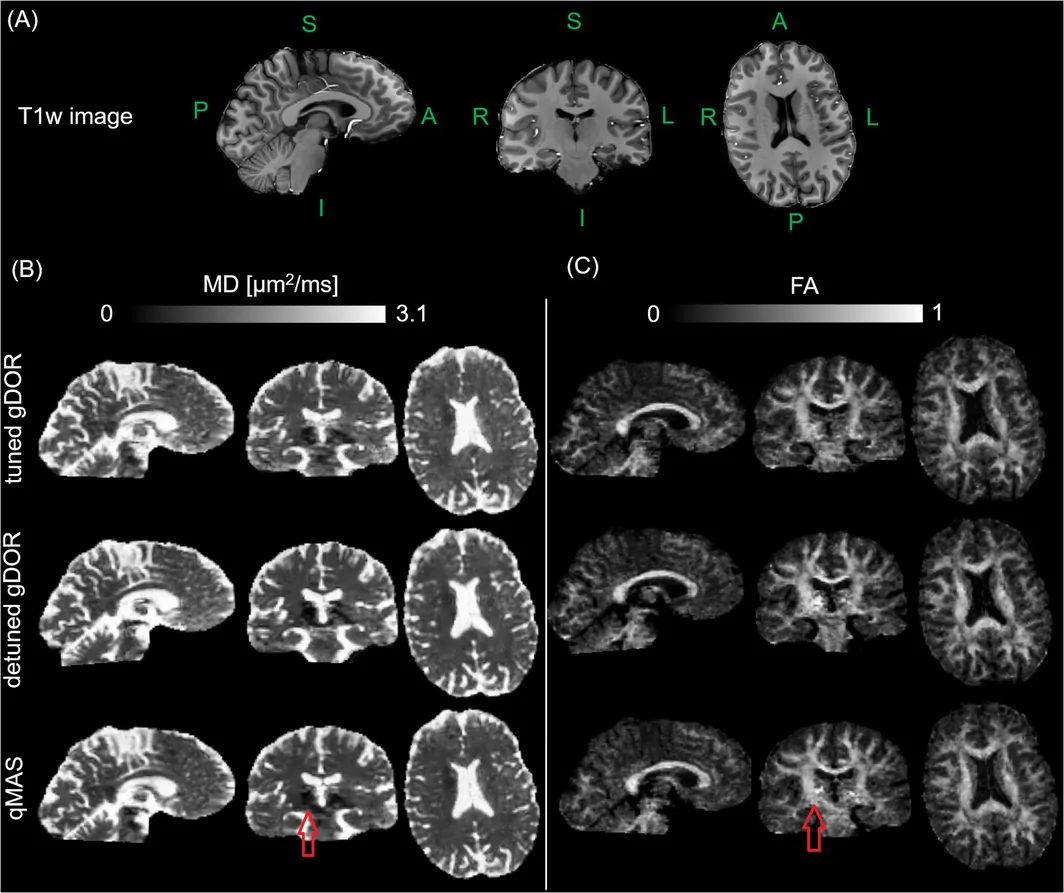
(A) Sagittal, coronal and transversal view of the T1w image of subject one. The orientation of the FOV, placed in green, is the same for all images in the figure. (B, C) Sagittal, coronal and transversal view of the macroscopic QTI metrics (MD: mean diffusivity, FA: fractional anisotropy) in structural space for the three sequences “tuned gDOR”, “detuned gDOR” and “qMAS.” The MD is slightly higher for tuned gDOR than for detuned gDOR and qMAS across the white matter, whereas for the FA there is no strong difference between the sequences visible. Red arrows show low MD and high FA in the region of the pallidum and caudate. This is the case for all sequences.

**FIGURE 7:**
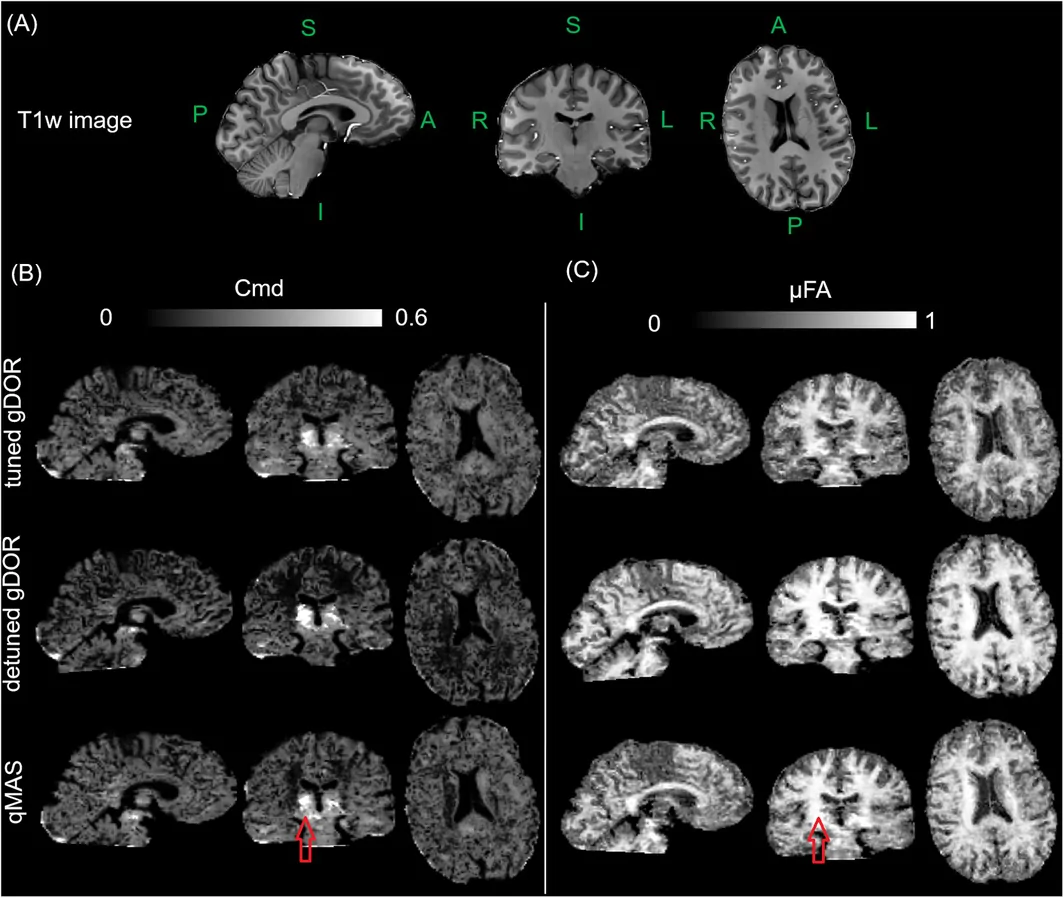
(A) Sagittal, coronal and transversal view of the T1w image of subject one. The orientation of the FOV, placed in green, is the same for all images in the figure. (B, C) Sagittal, coronal and transversal view of the microscopic QTI metrics (Cmd: isotropic variance, μFA: microscopic fractional anisotropy) in structural space for the three sequences “tuned gDOR”, “detuned gDOR” and “qMAS”. The μFA metric increases in its value from tuned gDOR over qMAS to detuned gDOR across the whole brain, whereas the Cmd decreases in the same way. Red arrows show high Cmd and high μFA in the region of the pallidum and caudate. This is the case for all sequences.

Differences between sequences were more enhanced for the region of the ACR (Table 2B and Figure 8) than for the splenium (Table S3 and Figure S7). The MD was higher for tuned gDOR compared to detuned gDOR and qMAS, whereas the Cmd was the highest for tuned gDOR, followed by qMAS and detuned gDOR. In contrast to FA, μFA varied considerably and was lowest for tuned gDOR, followed by qMAS and detuned gDOR. These differences for MD, Cmd, and μFA were tested as significant with regards to the mixed‐effects model statistics (small confidence intervals in Table 2B). The test and retest measurements of subject one showed a similar trend of the metric comparison between sequences.

## 5 Discussion

We investigated the design of QTI waveforms in terms of their encoding spectra for human in vivo applications. The spectra determine if QTI metrics specific to a centroid frequency can be measured (centroid frequency approximation). If the approximation is met, the Gaussian‐phase approximated signal S(ω) meets the signal defined by the tissue's DTD at the centroid frequency S(ω_c_). The approximation was first validated using idealized QTI waveforms and a comparison of analytical signal equations, and then applied in vivo by accordingly designing realistic and suitable QTI schemes. First, the theoretical investigations based on the idealized waveforms are discussed with respect to the frequency‐dependent properties of the encoded b‐tensors.

### 5.1 Same Centroid Frequency and Same Bandwidth Across b‐Tensor Shapes

Different encoding spectra with the same ω_c_ and bandwidth were designed. In that case, both tuning and spectral isotropy have to be applied as spectral anisotropy of the PTE waveform leads to different ω_c_ between the total PTE and LTE waveform. The approximation is expected to improve with more narrowly distributed encoding spectra around ω_c_. However, this depends on the tissue type's DTD.

A DTD consisting of one isotropic tensor with diffusivity following the power law ω^1^ does not lead to an approximation error for both a small and a large bandwidth due to the linear behavior of the power law (Figure 3B). In contrast, one anisotropic tensor with diffusivities following the power laws ω^1^ and ω^0.5^ leads to a larger deviation from the centroid frequency metrics due to the changing curvature of the power law ω^0.5^, especially at frequencies close to zero (Figure 3C and Figure S6: Cmd: 0.06%, μFA: 6%).

Adding parallel fibers to the anisotropic tensor results in growing differences of the diffusivities perpendicular to the principal fiber orientation with frequency. This is due to the intra‐axonal perpendicular diffusivity not being detectable for the investigated diffusion times [11], whereas the perpendicular diffusivity of the extra‐axonal compartment increases linearly with frequency [8, 11]. Therefore, the higher order terms in the cumulant expansion increase, and the cumulant signal S_c_(ω_c_) deviates from the signal at centroid frequency S(ω_c_) (Figure 3D and Figure S5). We refer to this as the truncation error of the cumulant expansion. Crossing instead of parallel fibers increase the truncation error as the diffusion differs more strongly across directions, influencing more diffusion volumes with different b‐tensor eigenbasis (Figure 3E). As a result, the truncation error becomes sufficiently large that a larger bandwidth no longer yields greater deviations from the centroid frequency metrics than a smaller bandwidth. Still, the deviations are small (Figure 5A, Cmd: 4%–8%, μFA: 3%–6%).

### 5.2 Different Centroid Frequency and Same Bandwidth Across b‐Tensor Shapes

Encoding spectra across b‐tensor shapes can differ in terms of their centroid frequencies, leading to a combined frequency as the mean of their centroid frequencies.

Lasič et al. [29, 30] showed that a varying spectral trace across b‐tensor shapes samples different MD across encodings, leading to over‐ or underestimated TVE metrics in ex vivo rat heart [29]/brain [30] as well as in the prostate [31]. This work builds up on that idea by comparing the over‐and underestimated metrics to the metrics at the combined frequency in case of human brain tissue. It is shown (Figure 4) that if PTE/LTE waveforms sample smaller/larger frequencies than the combined frequency, the signal level of the signal S(ω) of the PTE volumes is much larger than the signal level of the signal S(ω_c_) at the combined frequency due to a decreased MD at smaller frequencies and the other way around for the LTE volumes. This approximation error of sampling at the combined frequency enlarges with increased MD frequency‐dependence. Therefore, the error is most pronounced for the isotropic tensor with the strongly increasing power law ω^1^ and less pronounced for the DTD including crossing fibers with the slower increasing power law ω^0.5^ (Figure 3B,E).

The difference of the signal level of the signal S(ω) across b‐tensor shapes gets wrongly interpreted, leading to large deviations from the metrics at combined frequency. For the macroscopic metrics, the deviation stays below 8% in the crossing fiber region (Figures 4B,C and 5). As LTE volumes outnumber PTE volumes twofold, the direction of the sequence MD deviation is predominantly determined by the LTE volumes. For the microscopic metrics, small differences in the signal levels for the different b‐tensor shapes result in erroneous Cmd and μFA values as pointed out in Ref. [29, 30, 31]. Omitting positivity constraints on **d** and **C** could result in microscopic metrics outside of their defined boundaries (Figure 4B,C). Including constraints (Figure 5), lower frequencies in the PTE volumes than the combined frequency over‐/underestimate Cmd/μFA (Cmd: 90%, μFA: −20%) and vice versa for the lower frequencies in the LTE volumes (Cmd: −90%, μFA: 37%).

In the future, it should be tested how the numbers of PTE and LTE volumes impact deviations from centroid frequency metrics. Here, we started from an optimized protocol [45] and showed that matching encoding spectra (tuning and spectral isotropy) result in metrics closer to the combined (centroid) frequency.

### 5.3 Different Bandwidth Across b‐Tensor Shapes

Similar to spectra with different ω_c_, b‐tensors with different bandwidths encode different diffusivity strengths, causing signal differences and deviations from the combined frequency (Figure 5). However, in comparison to the deviation of different ω_c_ across b‐tensor shapes, these deviations are negligible.

### 5.4 In Vivo Validation

QTI metrics were successfully measured with a multiband spiral sequence at 7T for three sequences differing in encoding spectra (Figures 6 and 7). For the given sequence parameters (TE = 120 ms, volume TR > 5 s) and T2 values of WM (3T: 62 ms, 7T: 45 ms) [73] the SNR at 7T is approximately 1.95 times higher compared to 3T assuming a factor of B0^1.65^ (Ref. [74]). The measurement would greatly benefit from a high‐performance gradient system [75, 76, 77, 78] as this would drastically reduce the length of the QTI waveforms and result in lower echo times. However, such a system was not available. Additionally, parts of the GM, such as the pallidum and caudate, contain lower SNR than WM due to the combination of iron content and diffusion behavior [79], leading to strongly biased QTI metrics in these regions.

In vivo, for the ACR (Figure 8 and Table 2) and the splenium (Figure S7 and Table S3), tuned gDOR leads to higher MD and Cmd and lower μFA values, but similar FA values compared to the other two sequences. The significant strength of the differences between sequences (Table 2) meets the results of the simulated crossing fiber region.

**FIGURE 8:**
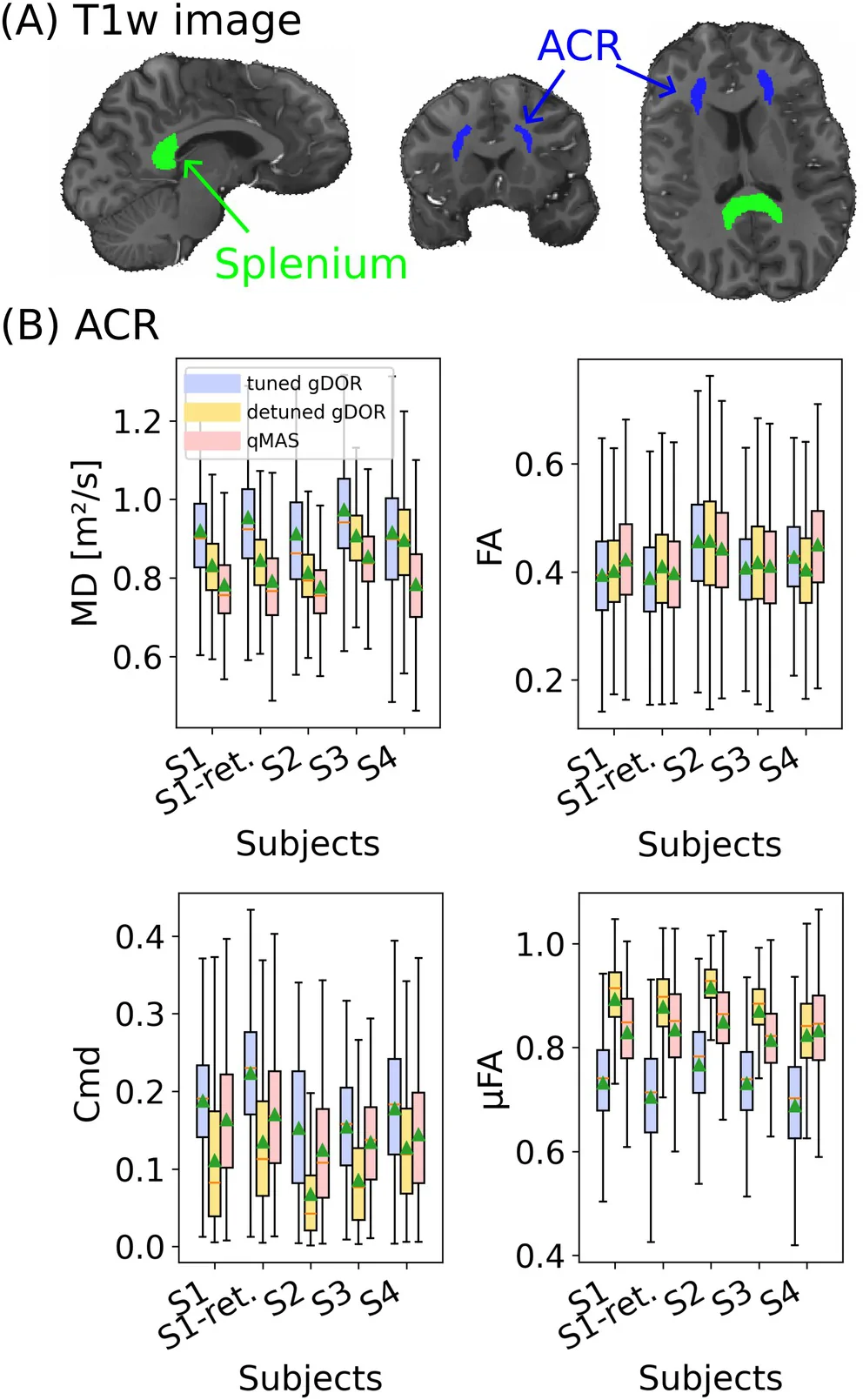
(A) Sagittal, coronal and transversal view of the T1w image of subject one, overlayed with the region of the splenium in green and the region of the anterior corona radiata (ACR) in blue. (B) Metrics across the anterior corona radiata of the four acquired subjects including the retest measurement of subject 1, denoted as “S1‐ret.” The red lines mark the mean and the triangles mark the median value.

Analytical spectra, created by results from literature [8, 9, 10, 11, 12], were sought to resemble the time‐dependent diffusion in the ACR. As the gradient waveforms used for human in vivo imaging contain small frequencies (ω_c_ < 18 Hz), the analytical spectra defined in the low frequency regime could be used as the ground‐truth effective diffusivity. The MD differs between intra‐ and extra‐axonal spaces because intra‐axonal radial diffusivity remains near zero even at higher frequencies, whereas extra‐axonal diffusivity increases linearly with frequency perpendicular to the fibers, increasing Cmd (Figures 4C and 5B). MD is increased as well as parallel and perpendicular diffusion in each space increase with frequency [27]. Crossing fibers decrease the FA due to orientational averaging of the diffusion tensor. Though the anisotropy of each of the tensors of the DTD increases with increased frequency, the denominator in the formula for FA and μFA contains the increased diffusivity, which results in a decrease of FA and μFA with increased frequency [27, 28]. However, the simulated analytical spectra only considered fitting values for the diffusion spectra of the ACR. In vivo, sequence‐related differences were greater in the ACR than in the splenium and may result from the larger extra‐axonal volume fraction in the crossing fiber region. Due to CSF contamination bias in the region of the splenium [12] and the need to account for an orientation dispersion inside a fiber bundle in the simulated DTD to yield an FA below one, only the ACR was simulated.

According to simulation (Table 2A), tuned gDOR has the most alike PTE and LTE spectra and therefore leads to metrics closer to metrics at the combined frequency (Cmd: −18%, μFA: 14%) compared to detuned gDOR having larger frequencies for PTE than LTE and therefore strongly under‐/overestimating Cmd/μFA (Cmd: −94%, μFA: 38%). The sequence qMAS falls between the gDOR sequences in terms of deviation (Cmd: −52%, μFA: 19%), as the LTE has lower frequencies than the spectrally anisotropic PTE, but not to that amount of detuned gDOR. The encoding spectrum of tuned gDOR is shifted towards larger frequencies, increasing sensitivity to the higher terms. As tuned gDOR contains the largest combined frequency, MD is largest, followed by detuned gDOR and qMAS.

As demonstrated, encoding spectra with same ω_c_ and bandwidth lead to minimal deviation from the centroid frequency metrics. Here, this was enabled with the optimized tuned gDOR sequence, ensuring not overstepping scanner limits and a reasonable echo time. Despite the two peaks in the encoding spectrum instead of one, the approximation error remains unchanged compared to one peak in the encoding spectrum for same bandwidth. The larger bandwidth (22.7 Hz) does not lead to additional bias due to the truncation error. However, due to a slight mismatch of LTE and PTE spectra in the optimization, the tuned gDOR leads still to slight deviations from the centroid frequency metrics (Table 2A: Cmd: −18%, μFA: 14%). An improvement would be to use the OGSE sequence from Figure S1C to match precisely PTE and LTE spectra. However, as PTE waveforms are set to the maximum gradient amplitude for minimal TE, tuning the LTE would result in overstepping the maximum gradient amplitude and therefore, the LTE waveform required separate optimization.

Care must be taken for in vivo measurements as scanner physical phenomena could lead to misinterpretation of the results in terms of time‐dependence. However, as differences of the metrics between the three sequences are prominent across the whole brain, they most likely do not originate from flow and partial‐volume effects of CSF [80]. B0/B1 inhomogeneities, background‐gradients or gradient‐nonlinearity effects would lead to local instead of global differences in the calculated metrics. Additionally, concomitant field effects are relatively low at 7T and eddy current effects were addressed by using the GTF in the b‐tensor computation. We also rule out water exchange as a major influence on the metrics, given the slow exchange rates in healthy white matter [81]. We observed both a signal and a frequency drift during diffusion measurements at high gradient duty cycle, which could lead to incorrect diffusion metrics and underestimated restriction [61]. While signal drift was corrected retrospectively [59] (Figure S8), the frequency drift can lead to reduced fat suppression for later acquired volumes [58]. However, we observed no fat artifacts in later acquired volumes. Finally, no vibration‐related signal attenuation resulting from the diffusion encoding was observed in any sequence (Figure S9), indicating minimal metric bias.

In summary, the simulations demonstrated that short‐range disorder induces time dependence in QTI metrics in human brain tissue when using the low‐frequency diffusion encoding spectra typical of in vivo imaging. Idealized waveforms with matched centroid frequency and bandwidth satisfied the centroid frequency approximation. In contrast to detuned gDOR and qMAS, tuned gDOR was optimized to match the encoding spectra of the idealized waveforms while considering hardware constraints. Despite a broadened spectral peak in the encoding spectra caused by the hardware constraints, this optimization enabled in vivo measurement of QTI metrics that were close to those obtained at the centroid frequency.

## 6 Conclusion

QTI waveforms were designed which ensure matching encoding spectra across b‐tensor shapes, including both tuning and spectral isotropy, and encoding spectra distributed around a centroid frequency. This enables probing QTI metrics at this specific frequency in complex tissue as in the human brain. Conversely, metrics of different encoding spectra across b‐tensor shapes deviate from centroid‐frequency metrics by a factor depending on the tissue's diffusivity, strongly over‐ or underestimating microscopic metrics. Because of scanner and timing constraints, spectra of the designed realistic waveforms are more broadly distributed around the centroid frequency. In simulated WM, however, truncation of the cumulant expansion causes spectra with different bandwidths to yield similar small deviations from the centroid frequency metrics.

## Funding

This work was supported by the European Union Horizon 2020 Research and Innovation Program (885876).

## Conflicts of Interest

The author Svenja Niesen declares no potential conflict of interests. The author Marten Veldmann declares no potential conflict of interests. The author Ali Ajouz declares no potential conflict of interests. Ali Ajouz is an employee of Siemens Healthineers AG and UKSH. The author Tony Stöcker declares no potential conflict of interests.

## Supporting information


**Table S1:** Optimization parameters for tuned (A, B) and detuned gDOR (A, C). Td: duration of the diffusion waveform including the refocusing duration. t180: refocusing duration. up1/plateau1/down1/ampl1 (up2/plateau2/down2/ampl2): ramp up time/flat top time/ramp down time/amplitude of the first (second) trapezoid. The total angle of rotation ΔΨ_2_ and n are the same as in Jiang et al., *Magn. Reson*., 2023.
**Table S2:** Optimization parameters for qMAS.
**Table S3:** Mean and mixed‐effects model statistics in the splenium demonstrate the highest MD for tuned gDOR, followed by detuned gDOR and qMAS, similar FA values and big differences between the gDOR sequences for Cmd and μFA.
**Figure S1:** (A) Tuned gDOR sequences, used for the in vivo measurements, including PTE and LTE waveforms from Table S1A,B. (B) Circular polarized oscillating gradient spin echo (CP‐OGSE) sequence from Lundell et al., MRM, 2015. (C) Oscillating gradient spin echo (OGSE) sequence. (A–C) Td: total waveform duration, *f*_*c*_: centroid frequency, Δ*f*: spectral bandwidth.
**Figure S2:** Crushing moments along each Cartesian axis of the scanner for each diffusion volume. Sequence: tuned gDOR.
**Figure S3:** Crushing moments along each Cartesian axis of the scanner for each diffusion volume. Sequence: detuned gDOR.
**Figure S4:** Crushing moments along each Cartesian axis of the scanner for each diffusion volume. Sequence: qMAS.
**Figure S5:** Relative mean absolute errors RMAE(S(ω), S(ω_c_)) to investigate the truncation error of the cumulant expansion are shown in a heat map for the 16 combinations of the idealized waveforms from Table 1 (pink markings in Figure 1D). The first arrow defines *f*_*c*_, the second Δ*f* and solid lines define PTE and dashed lines LTE waveforms. Each combination corresponds to one cell, divided into two triangles. The RMAE in the lower triangle includes only the LTE volumes, which lead to a value of less than 0.3 for the inner scalar product of their gradient direction with the principal fiber orientation (0, 0, 1)^T^. The upper triangle includes the other LTE volumes (see demonstration on the right side of the Figure). The error from truncation of the cumulant expansion increases with an increasement in diffusivity differences in the tensor distribution and as the extra‐axonal tensor differs from the parallel fiber tensors in terms of the diffusivity perpendicular to the principal fiber orientation, the cumulant error increases for diffusion weighting along the perpendicular direction.
**Figure S6:** Relative errors (RE) of the QTI metrics for the simulated DTD with one anisotropic tensor. Note, for QTI sequences with matching PTE and LTE encoding spectra (diagonals, marked by a green rectangular shape) the metric agrees more with the metric at the centroid frequency if the spectral bandwidth is decreased.
**Figure S7:** (A) Sagittal, coronal and transversal view of the T1w image of subject one, overlayed with the region of the splenium in green and the region of the anterior corona radiata (ACR) in blue. (B) Metrics across the splenium of the four acquired subjects including the retest measurement of subject 1, denoted as “S1‐ret.” The red lines mark the mean and the triangles mark the median value.
**Figure S8:** The signal drift for all three sequences is shown for subject one as an example (code for the creation of the figures was adapted from Vos et al., MRM, 2017). On the left side, a transversal slice demonstrates the classification of the 10 clusters.
**Figure S9:** The median of the signal intensity within the splenium and in the gray matter (GM) is calculated over diffusion volumes with the *x*‐, *y*‐ and *z*‐component of the third eigenvector (bvec). Only the LTE diffusion volumes and volumes with the maximum *b*‐value are considered. The figure is adapted from figure 2 from Gallichan et al., Hum. Brain Mapp., 2010. On the left side, a transversal slice of the mean non‐diffusion weighted image of the qMAS sequence is overlayed with the binary masks of the splenium (red) and the GM (blue). The data corresponds to the measurement of subject one. Whereas the splenium shows directional‐dependent signal decay along the *x*‐axis and partly along the *z*‐axis of the scanner due to diffusion along the fiber orientations, the GM does not show directional‐dependent signal decay. As described by Gallichan et al., a signal decay, that starts at a component value of approximately 0.75 illustrates signal decay due to vibration. The three investigated sequences do not exhibit this behavior, which indicates low to vanishing signal decay due to vibration.

## Data Availability

The sequence designed with Pulseq can be found in the following openly available Github repository: https://github.com/mrphysics-bonn/spiral-tve/tree/paper.
