# Supplementary material for: Human In Vivo Validation of Frequency‐Dependent QTI
Source: Magn Reson Med. 2026 Aug 13;96(6):2642–59. doi: 10.1002/mrm.70529 (PMC13619846; doi:10.1002/mrm.70529)
Supplement: Supplementary file 1 [file MRM-96-2642-s001.docx]

**Supporting Information Table S1**: Optimization parameters for tuned (A,B) and detuned gDOR (A,C). Td: duration of the diffusion waveform including the refocusing duration. t180: refocusing duration. up1/plateau1/down1/ampl1 (up2/plateau2/down2/ampl2): ramp up time/flat top time/ramp down time/ amplitude of the first (second) trapezoid. The total angle of rotation $\Delta\Psi_{2}$ and n are the same as in Ref. ^1^.

(A) PTE (spectral isotropy)

(C) LTE (detuned)

(B) LTE (tuned)

|  | Td [s]  t180 [s] | up1 [ms]  plateau1 [ms]  down1 [ms] | time between [ms] | up2 [ms]  plateau2 [ms]  down2 [ms] | ampl1 [mT/m]  ampl2 [mT/m] | $\Delta\Psi_{2}$ [rad]  n |
| --- | --- | --- | --- | --- | --- | --- |
| (A) | 111ms  11.5ms | 0.4  4.42  5.83 | 28.82 | 6.92  2.96  0.4 | 53.5  54.7 | 9.2666  0 |
| (B) | 111ms  11.5ms | 3.31  6.17  6.68 | 21.25 | 7.25  4.68  0.4 | 67.983  69.975 | 3.4257  1.8988 |
| (C) | 111ms  11.5ms | 0.39  3.6  7.03 | 2.57 | 13.20  9.72  13.24 | 67.417  5.0665 | 5.5371  0 |

^1^ Jiang H, Svenningsson L, Topgaard D. Multidimensional encoding of restricted and anisotropic diffusion by double rotation of the *q* vector. *Magn Reson*. 2023;4(1):73-85. doi:10.5194/mr-4-73-2023

**Supporting Information Table S2**: Optimization parameters for qMAS.

| spacing $\Delta$ [ms] | delta $\delta$ [ms] | Maximum gradient amplitude G_T_ [mT/m] | Maximum gradient slew rate [T/m/s] |
| --- | --- | --- | --- |
| 77.1 | 8.86 | 57.97 | 178.60 |

**Supporting Information Table S3**: Mean and mixed-effects model statistics in the splenium demonstrate the highest MD for tuned gDOR, followed by detuned gDOR and qMAS, similar FA values and big differences between the gDOR sequences for Cmd and µFA.

| **Splenium in vivo** | Mean $\pm$ SD | | | absolute difference  relative difference [%]  95% CI (relative difference [%]) | | |
| --- | --- | --- | --- | --- | --- | --- |
|  | tuned gDOR | detuned gDOR | qMAS | qMAS > detuned gDOR | tuned gDOR > detuned gDOR | qMAS > tuned gDOR |
| MD | 1.04$\pm$0.14 [µm^2^/ms] | 0.99$\pm$0.08 [µm^2^/ms] | 0.92$\pm$0.09 [µm^2^/ms] | -0.07 [µm^2^/ms]  -6.85  [-7.16,-6.53] | 0.05 [µm^2^/ms]  5.35  [5.04,5.66] | -0.12 [µm^2^/ms]  -13.09  [-13.76,-12.43] |
| FA | 0.60$\pm$0.04 | 0.60$\pm$0.05 | 0.60$\pm$0.04 | -0.0003  -0.05  [-0.33,0.24] | -0.004  -0.64  [-0.92,-0.35] | 0.004  0.59  [0.02,.1.16] |
| Cmd | 0.17$\pm$0.07 | 0.13$\pm$0.05 | 0.18$\pm$0.04 | 0.05  42.60  [42.14,43.07] | 0.04  33.51  [33.04,33.98] | 0.01  6.38  [5.72,7.03] |
| µFA | 0.79$\pm$0.07 | 0.88$\pm$0.05 | 0.83$\pm$0.04 | -0.04  -5.06  [-5.20,-4.92] | -0.09  -10.16  [-10.30,-10.02] | 0.04  5.36  [5.07,5.66] |

**Supporting Information Figure S1**: (A) Tuned gDOR sequences, used for the in vivo measurements, including PTE and LTE waveforms from Supporting Table S1A-B. (B) Circular polarized oscillating gradient spin echo (CP-OGSE) sequence from Ref. ^2^. (C) Oscillating gradient spin echo (OGSE) sequence. (A-C) Td: total waveform duration, fc: centroid frequency, Δf: spectral bandwidth.

^2^ Lundell H, Sønderby CK, Dyrby TB. Diffusion weighted imaging with circularly polarized oscillating gradients: Circularly Polarized Diffusion Encoding. *Magn Reson Med*. 2015;73(3):1171-1176. doi:10.1002/mrm.25211

**Supporting Information Figure S2**: Crushing moments along each Cartesian axis of the scanner for each diffusion volume. Sequence: tuned gDOR.

**Supporting Information Figure S3**: Crushing moments along each Cartesian axis of the scanner for each diffusion volume. Sequence: detuned gDOR.

**Supporting Information Figure S4**: Crushing moments along each Cartesian axis of the scanner for each diffusion volume. Sequence: qMAS.

**Supporting Information Figure S5**: Relative mean absolute errors RMAE(S(ω),S(ω_c_)) to investigate the truncation error of the cumulant expansion are shown in a heat map for the 16 combinations of the idealized waveforms from Table 1 (pink markings in Figure 1D). The first arrow defines fc, the second Δf and solid lines define PTE and dashed lines LTE waveforms. Each combination corresponds to one cell, divided into two triangles. The RMAE in the lower triangle includes only the LTE volumes, which lead to a value of less than 0.3 for the inner scalar product of their gradient direction with the principal fiber orientation (0,0,1)^T^. The upper triangle includes the other LTE volumes (see demonstration on the right side of the Figure). The error from truncation of the cumulant expansion increases with an increasement in diffusivity differences in the tensor distribution and as the extra-axonal tensor differs from the parallel fiber tensors in terms of the diffusivity perpendicular to the principal fiber orientation, the cumulant error increases for diffusion weighting along the perpendicular direction.

**Supporting Information Figure S6**: Relative errors (RE) of the QTI metrics for the simulated DTD with one anisotropic tensor. Note, for QTI sequences with matching PTE and LTE encoding spectra (diagonals, marked by a green rectangular shape) the metric agrees more with the metric at the centroid frequency if the spectral bandwidth is decreased.

**Supporting Information Figure S7**: (A) Sagittal, coronal and transversal view of the T1w image of subject one, overlayed with the region of the splenium in green and the region of the anterior corona radiata (ACR) in blue. (B) Metrics across the splenium of the four acquired subjects including the retest measurement of subject 1, denoted as ‘S1-ret.’. The red lines mark the mean and the triangles mark the median value.

**Supporting Information Figure S8**: The signal drift for all three sequences is shown for subject one as an example (Code for the creation of the Figures was adapted from Ref. ^3^). On the left side, a transversal slice demonstrates the classification of the ten clusters.

^3^ Sjoerd Vos (2025). Signal drift correction for diffusion MRI data (https://de.mathworks.com/matlabcentral/fileexchange/55008-signal-drift-correction-for-diffusion-mri-data), MATLAB Central File Exchange. Last visit: 30th. November 2025.

**Supporting Information Figure S9**: The median of the signal intensity within the splenium and in the gray matter (GM) is calculated over diffusion volumes with the x-,y- and z-component of the third eigenvector (bvec). Only the LTE diffusion volumes and volumes with the maximum b-value are considered. The Figure is adapted from Figure 2 from Ref. ^4^. On the left side, a transversal slice of the mean non-diffusion weighted image of the qMAS sequence is overlayed with the binary masks of the splenium (red) and the GM (blue). The data corresponds to the measurement of subject one. Whereas the splenium shows directional-dependent signal decay along the x-axis and partly along the z-axis of the scanner due to diffusion along the fiber orientations, the GM does not show directional-dependent signal decay. As described by Ref. ^4^, a signal decay, that starts at a component value of approximately 0.75 illustrates signal decay due to vibration. The three investigated sequences do not exhibit this behavior, which indicates low to vanishing signal decay due to vibration.

^4^ Gallichan D, Scholz J, Bartsch A, Behrens TE, Robson MD, Miller KL. Addressing a systematic vibration artifact in diffusion‐weighted MRI. *Hum Brain Mapp*. 2009;31(2):193-202. doi:10.1002/hbm.20856
